# *Shigella* Serotypes Associated With Carriage in Humans Establish Persistent Infection in Zebrafish

**DOI:** 10.1093/infdis/jiad326

**Published:** 2023-08-09

**Authors:** Vincenzo Torraca, Dominik Brokatzky, Sydney L Miles, Charlotte E Chong, P Malaka De Silva, Stephen Baker, Claire Jenkins, Kathryn E Holt, Kate S Baker, Serge Mostowy

**Affiliations:** Department of Infection Biology, London School of Hygiene and Tropical Medicine, London, United Kingdom; School of Life Sciences, University of Westminster, London, United Kingdom; Department of Infectious Diseases, School of Immunology and Microbial Sciences, King’s College London, London, United Kingdom; Department of Infection Biology, London School of Hygiene and Tropical Medicine, London, United Kingdom; Department of Infection Biology, London School of Hygiene and Tropical Medicine, London, United Kingdom; Clinical Infection, Microbiology, and Immunology, Institute of Infection, Veterinary and Ecological Sciences, University of Liverpool, Liverpool, United Kingdom; Clinical Infection, Microbiology, and Immunology, Institute of Infection, Veterinary and Ecological Sciences, University of Liverpool, Liverpool, United Kingdom; Department of Medicine, School of Clinical Medicine, University of Cambridge, Cambridge, United Kingdom; Gastrointestinal Bacterial Reference Unit, UK Health Security Agency, London, United Kingdom; Department of Infection Biology, London School of Hygiene and Tropical Medicine, London, United Kingdom; Department of Infectious Diseases, Central Clinical School, Monash University, Melbourne, Australia; Clinical Infection, Microbiology, and Immunology, Institute of Infection, Veterinary and Ecological Sciences, University of Liverpool, Liverpool, United Kingdom; Department of Genetics, University of Cambridge, Cambridge, United Kingdom; Department of Infection Biology, London School of Hygiene and Tropical Medicine, London, United Kingdom

**Keywords:** O-antigen, persistent infection, *Shigella flexneri*, *Shigella sonnei*, zebrafish

## Abstract

*Shigella* represents a paraphyletic group of enteroinvasive *Escherichia coli*. More than 40 *Shigella* serotypes have been reported. However, most cases within the men who have sex with men (MSM) community are attributed to 3 serotypes: *Shigella sonnei* unique serotype and *Shigella flexneri* 2a and 3a serotypes. Using the zebrafish model, we demonstrate that *Shigella* can establish persistent infection in vivo. Bacteria are not cleared by the immune system and become antibiotic tolerant. Establishment of persistent infection depends on the O-antigen, a key constituent of the bacterial surface and a serotype determinant. Representative isolates associated with MSM transmission persist in zebrafish, while representative isolates of a serotype not associated with MSM transmission do not. Isolates of a *Shigella* serotype establishing persistent infections elicited significantly less macrophage death in vivo than isolates of a serotype unable to persist. We conclude that zebrafish are a valuable platform to illuminate factors underlying establishment of *Shigella* persistent infection in humans.

## Teaser

Torraca et al use the zebrafish model to study *Shigella* persistent infection. The authors show that the *Shigella* O-antigen is essential to establish persistent infection in vivo and that the carriage-serotype associations observed in humans are also observed in zebrafish.

Shigellosis is a diarrheal disease caused mainly by *Shigella sonnei* and *Shigella flexneri* [1]. Shigellosis is normally managed with rehydration therapy and antibiotics (fluoroquinolones, extended-spectrum β-lactams, azithromycin [2]). However, antimicrobial resistance is widespread, and *Shigella* is considered an antimicrobial resistance priority pathogen [3, 4].

Infection from *Shigella* is viewed to be self-limiting and short-lived. In high-income countries, sexual transmission of shigellosis in men who have sex with men (MSM) is the major route of domestic dissemination. In a recent retrospective cohort study performed in the United Kingdom, *Shigella* was longitudinally sampled from patients. In a subset of cases, a small single-nucleotide polymorphism distance (<8 nucleotides) between serial isolates was determined [5], which is suggestive of persistent carriage. The MSM community, where serial detection has been frequently identified, is also at increased risk of HIV and reinfections from sexual partners. However, symptomatic and asymptomatic carriage of *Shigella* has been reported in children and the general adult population in different settings [6–10]. Pathogens recognized to establish persistent infection represent a major public health burden; prominent examples include *Helicobacter pylori*, *Salmonella enterica*, and *Mycobacterium tuberculosis* [11–14]. Persistent infections are not completely cleared by the immune system and are recalcitrant to antimicrobial therapy [15]. These infections can become asymptomatic and promote dissemination upon disease reactivation.

The zebrafish is a widely adopted model and valuable to investigate infection by a variety of human bacterial pathogens, including enterobacteria (*Escherichia coli*, *Salmonella*, and *Shigella*) [16, 17]. Zebrafish larvae have an innate immune system highly homologous to that of humans, and their transparency enables high-resolution intravital imaging of fluorescently tagged immune cells and bacteria in vivo [16, 18]. Here, using a *Shigella*-zebrafish infection model, we discover that clinical isolates of *Shigella* can establish a persistent and antibiotic-tolerant infection in zebrafish for at least 6 days. Our results demonstrate a new role for *Shigella* O-antigen (O-Ag) variants in enabling persistent infection and highlight zebrafish as an animal model that can be used to investigate the surge of 3 dominant *Shigella* serotypes circulating in the MSM community [19–21].

## METHODS

### Bacterial Strains

Wild-type and genetically modified bacterial strains are detailed in Supplementary Table 1 and Supplementary Table 2. Parental strains were made fluorescent by electroporation of pFPV25.1 (GFP labeled) or pFPV-mCherry (mCherry labeled), conferring carbenicillin resistance. Exception was made for *S. sonnei* 373976, which was intrinsically resistant to high concentrations of carbenicillin (minimum inhibitory concentration > 8000 μg/mL). Bacterial mutant strains were previously described [22].

### Zebrafish Model and Animal Experimentation Guidelines

Animal experiments were approved by the Home Office (PPL P4E664E3C) and performed following the Animals (Scientific Procedures) Act 1986. Wild type AB zebrafish were used for survival assays and colony-forming unit (CFU) quantification experiments. For experiments involving macrophage imaging, the transgenic line *Tg(mpeg1::Gal4-FF)*^gl25^/*Tg(UAS::LIFEACT-GFP)*^mu271^ was used. Eggs were obtained by natural spawning, and larvae were maintained at 28.5 °C in embryo medium (0.5× E2 medium supplemented with 0.5 ppm of methylene blue). For injections and live microscopy, larvae were anesthetized with 200 μg/mL tricaine (Sigma-Aldrich) in embryo medium. Infected larvae were monitored up to 6 days postinfection. Sex was not determined, as experiments were concluded before the zebrafish’s sexual development.

### Zebrafish Infection

Bacteria were cultured at 37 °C overnight in trypticase soy broth supplemented, when appropriate, with 100 μg/mL carbenicillin, diluted 50× in fresh medium, and grown to log phase. For inoculum preparation, bacteria were spun down, washed in phosphate-buffered saline (PBS), and resuspended to an OD_600_ of 2 in an injection buffer containing 2% polyvinylpyrrolidone (Sigma-Aldrich) and 0.5% phenol red (Sigma-Aldrich) in PBS. Unless otherwise specified, 1 nL of bacterial suspension (corresponding to approximately 1000 CFUs) was microinjected in the hindbrain ventricle of zebrafish larvae at 3 days postfertilization (dpf).

### Survival Assays and Bacterial Burden

Larvae failing to produce a heartbeat during a 30-second observation were considered nonviable. For larvae beyond 5 dpf, clinical scoring criteria were applied to identify humane endpoints. Briefly, larvae were discontinued when bacterial load was predictive of death in the following 24 hours (ie, hindbrain filled with bacteria and/or systemic dissemination) or when larvae were irresponsive to touch. These larvae were withdrawn from the experiment, euthanized, and considered nonviable at subsequent time points.

For CFU enumeration, larvae were washed in PBS, anesthetized, and mechanically homogenized in 200 μL (nonpersistent stages) or 40 μL (persistent stages) of PBS. Homogenates were serially diluted and plated onto trypticase soy agar containing 0.01% Congo red (Sigma-Aldrich). Only viable larvae were used for CFU analysis.

### Clonality Assay

Larvae were infected as previously specified but with an inoculum of GFP–*S. sonnei* and mCherry–*S. sonnei* in a 1:1 ratio. The ratio between the strains was determined by CFU plating at 0 hours postinfection (hpi) and 144 hpi. At 0 hpi, dilutions of samples were made to correct for differences in the occurrence of stochastic bottlenecks.

### Microscopy

For in vivo time-lapse imaging, larvae were immobilized in 1% low–melting point agarose. For high-resolution confocal microscopy, larvae were positioned in glass-bottom MatTek dishes (35 mm diameter), and imaging was performed with a Zeiss LSM 880 with Fast Airyscan and 20× or 40× water immersion objectives. Image files were processed with Fiji ImageJ. Colocalization percentage between macrophages and bacteria was determined from fluorescent images. Z-stacks encompassing bacterial signal were projected in a bidimensional image. The mCherry channel (*S. sonnei*) and the GFP channel (macrophages) were extracted and converted to binary masks. The percentage of mCherry area overlapping with the GFP area was calculated.

For imaging fixed samples, larvae were fixed in 2% paraformaldehyde in PBS at 4 °C overnight, followed by 2-times washing with PBS and 15 minutes of permeabilization with 1× proteinase K (Component H from Click-iT Plus TUNEL Assay C10619; Thermo Fisher Scientific) at room temperature. Larvae were then washed in PBS, stained with Hoechst 33342 at 1:500 in water for 30 minutes at room temperature, washed, and transferred to 60% (v/v) glycerol via gradient washes.

### Drug Treatments

Nalidixic acid (Nal) was added to embryo medium to reach the desired concentration. In pilot experiments, a range of concentrations was tested (0.5–4.0 μg/mL), and 1 μg/mL was the lowest that fully prevented bacterial growth and death of larvae upon infection with a lethal dose of *S. sonnei* 53G.

### Flow Cytometry and Caspase 1 Activity Quantification

A pool of 3 dpf *Tg(mpeg1::Gal4-FF)*^gl25^/*Tg(UAS::LIFEACT-GFP)*^mu271^ larvae were infected systemically with 10000 CFUs of mCherry–*S. sonnei* 53G, mCherry–*S. flexneri* M90T, or PBS containing mock solution. At 4 hpi, larvae were dissociated by treatment with 1 mL of 4% trypsin for 15 minutes at 28 °C. Single-cell dissociation was facilitated by mechanical disruption with a P1000 pipette after trypsin treatment. Dissociated cells were harvested by centrifugation (5 minutes, 800*g*, room temperature), washed in calcium-free PBS, separated by passage on a 4 μm cell strainer, and suspended in 500 μL of staining solution for active caspase 1 (FLICA 660 Caspase-1 Assay Kit, probe 660-YVAD-FMK, No. 9122; ImmunoChemistry Technologies) prepared per the manufacturer's guidelines. Upon staining, cells were pelleted, washed in PBS, and fixed in 4% paraformaldehyde overnight. For flow cytometry, cells were washed with 2 mL of PBS twice and resuspended in 300 μL of PBS. Active caspase 1 staining of single macrophages was measured on a LSRII (BD Biosciences) and data were analyzed with FlowJo version 10.7.1.

### DNA Extraction and Sequencing

Genomic DNA was extracted from overnight bacterial cultures with a MasterPure Complete DNA Purification Kit (Lucigen Corporation) according to the manufacturer's instructions. DNA concentration, purity, and quantity were assessed with NanoDrop spectrophotometry (DeNovix) and Qubit fluorometer (Invitrogen) according to the manufacturers’ instructions. Sequencing libraries were prepared with a ligation sequencing kit (SQK-LSK109; Oxford Nanopore Technologies) according to the manufacturer's instructions, with modifications to input DNA; specifically, amounts were increased at least 2-fold at the initial step. DNA libraries were sequenced with a MinION sequencer and FLO-MIN106 Flow Cell R9.4.1 (Oxford Nanopore Technologies).

### Assembly and Annotation

The fast5 read files generated from the MinION instrument were base called and demultiplexed with Guppy version 5.0 (Oxford Nanopore Technologies). Processed read files were filtered with Filtlong version 0.2.0 and assembled with Flye version 2.9 [23]. Racon version 1.5.0 [24] was used to polish contigs with nanopore reads, and Medaka version 1.6.1 (https://github.com/nanoporetech/medaka) was used to polish Racon polished contigs, with nanopore reads specifying the model r941_min_sup_g507. Polypolish version 0.5.0 was used to polish with Illumina reads, where available [25]. The quality and statistics of each assembly were evaluated with QUAST version 4.4.0 without a reference genome [26]. Genomes were annotated with Prokaryotic Genome Annotation Pipeline. The complete genome sequence data have been submitted to the National Center for Biotechnology Information and deposited at GenBank under BioProject PRJNA869897.

### Phylogenetic Tree Construction

Annotations of genomes and tree constructions were performed with Genome Annotation services made available by the Bacterial and Viral Bioinformatics Resource Centre (BV-BRC; https://www.bv-brc.org/) [27]. For genome annotation, parameters were set as follows: annotation recipe, bacteria/archaea; taxonomy name, *Shigella*. Phylogenetic trees were constructed with the codon tree–building method, and parameters were set as follows: number of genes, 1000; maximum allowed deletions, 1; maximum allowed duplications, 1. Data included complete genomes of *S. sonnei* and *S. flexneri* isolates (publicly available via the BV-BRC database as of November 2022) and draft genomes of all isolates sequenced/resequenced and tested in this study (Supplementary Table 1). In cases where *S. flexneri* serotype was unknown, this was predicted with ShigEiFinder [28]. Trees also include draft genomes for isolates reported to represent likely cases of carriage in humans with a distance between collected isolates of at least 10 days [29]. Since this data set represented pairs of isolates with a single-nucleotide polymorphism distance <8, only sequences from second isolates were used. Data were annotated, edited, and visualized with FigTree version 1.4.4. (http://tree.bio.ed.ac.uk/software/figtree/), iTOL (https://itol.embl.de/), and Inkscape version 1.2.2 (https://inkscape.org/).

### Quantification and Statistical Analysis

Statistical tests were performed with Prism 9 (GraphPad) or Microsoft Excel. Statistical significance of the survival curves was determined with the log-rank Mantel-Cox test. The difference in distribution at different time points in the clonality assay was determined by the chi-square test. In all other cases, statistical significance was determined with an unpaired 2-tailed *t* test or 1-way analysis of variance with Sidak correction. Analyses were performed on log_10_-transformed data for CFU counts. Data are represented as mean ± SEM.

## RESULTS

### *Shigella* Can Establish Persistent Infection in Zebrafish

**Figure 1.**
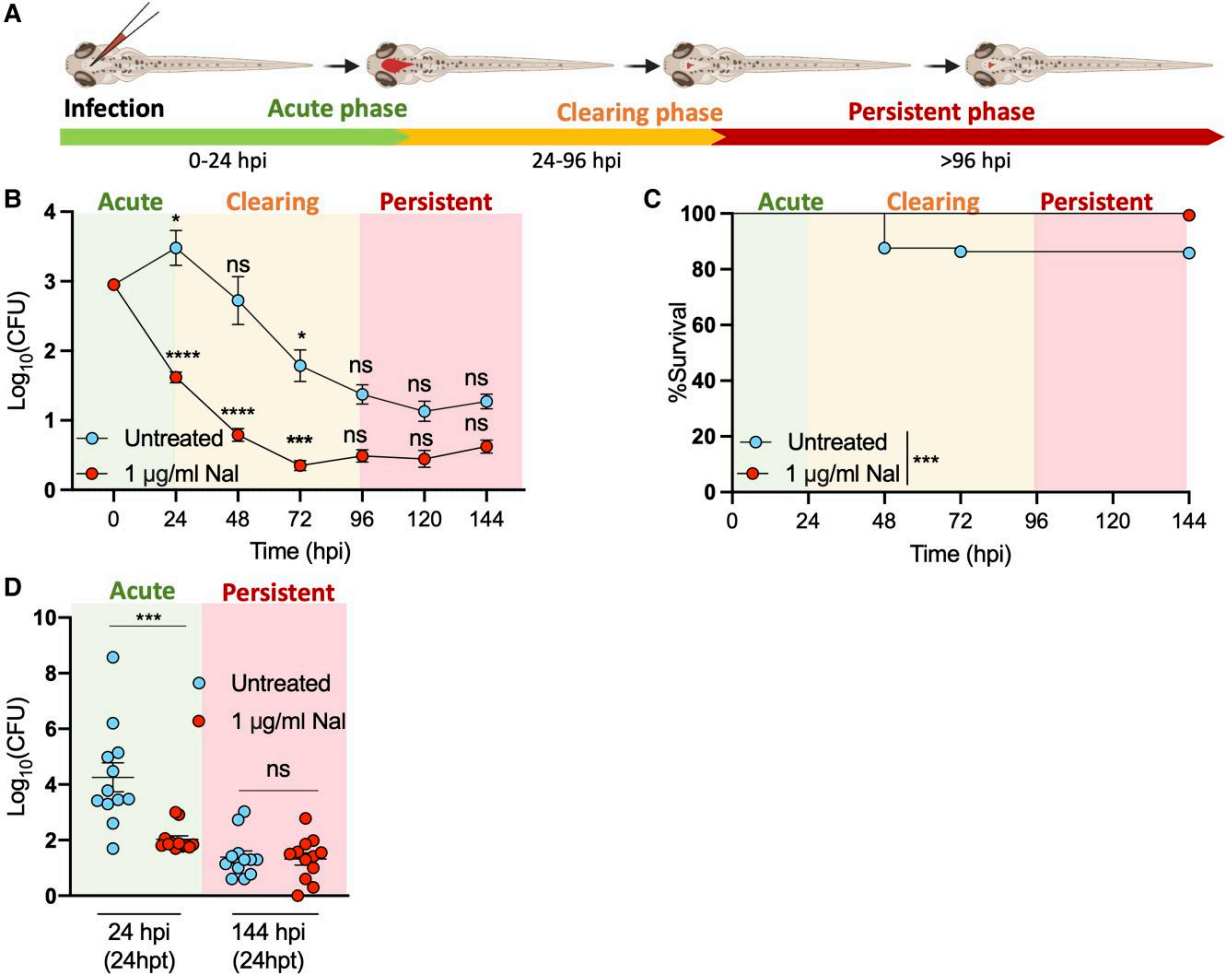
*Shigella* establishes persistent infection in zebrafish. *A*, Experimental diagram of *S. sonnei*–zebrafish infection. *S. sonnei* infection undergoes 3 phases: an acute phase (ie, increasing bacterial load), a clearing phase (ie, a steady decrease of bacterial load), and a persistent phase (ie, bacterial load does not further decrease). Diagram created with BioRender.com. *B* and *C*, CFU count and survival analysis from zebrafish larvae infected with *S. sonnei* 53G. Larvae were treated with the antibiotic Nal or left untreated. *S. sonnei* establishes a persistent infection in zebrafish, even during treatment with a therapeutic antibiotic dose. From 96 hpi, the bacterial load in larvae remains constant over consecutive days. *D*, CFU count from zebrafish larvae treated with Nal or left untreated during the acute and persistent infection stages. During the acute infection stage (0–24 hpi), *S. sonnei* is sensitive to antibiotic treatment, while during the persistent infection stage (120–144 hpi), *S. sonnei* becomes insensitive to antibiotic treatment. Data were analyzed at 24 hours posttreatment. Data: mean ± SEM. Statistics: *B*, unpaired *t* test on log_10_-transformed data against the previous time point; *C*, log-rank Mantel-Cox test; *D*, 1-way analysis of variance with Sidak correction. ns, *P* ≥ .05. *P < .05. ***P < .001. CFU, colony-forming unit; hpi, hours postinfection; hpt, hours posttreatment; Nal, nalidixic acid; ns, nonsignificant.

To test if *Shigella* can establish persistent infection in zebrafish, we injected a low dose of mCherry–*S. sonnei* 53G (approximately 1000 CFUs, characteristically eliciting approximately 20% host death by 72 hpi) in the hindbrain ventricle of zebrafish larvae at 3 dpf and assessed bacterial burden and host survival daily up to 144 hpi (Figure 1*A*–*C*, Supplementary Figure 1*A–C*, Supplementary Data set). Quantification of bacterial burden (Figure 1*B*) and linear regression analysis (Supplementary Figure 1*A*) indicated that infection progresses in 3 distinct phases: an acute phase characterized by bacterial replication (0–24 hpi), a clearing phase characterized by a significant decrease of bacteria at a constant rate (24–96 hpi), and a persistent phase where few bacteria (<5% of the initial bacterial load) are maintained over an extended period (96–144 hpi).

Persistent infections are often recalcitrant to antibiotic treatment. To test if the persistent bacterial population is tolerant to antibiotics, we established a therapeutic dose to treat *S. sonnei* infection of zebrafish larvae with Nal. Overall 1 μg/mL of Nal was the lowest tested dose that prevented the death of all larvae exposed to a lethal dose of *S. sonnei* (approximately 8000 CFUs, characteristically eliciting approximately 80% host death by 72 hpi), as well as bacterial proliferation within the host (Supplementary Figure 1*D, E*). In addition, 0.5 μg/mL of Nal was tested, but this was ineffective and unable to control host death or bacterial proliferation within the host. Higher doses (2 and 4 μg/mL) were able to prevent bacterial proliferation, but treatments led to more phenotypic aberrations in exposed larvae as compared with larvae treated with 1 μg/mL (infected and uninfected, Supplementary Figure 1*F*); it also led to death due to toxicity (Supplementary Figure 1*D*) rather than bacterial burden (Supplementary Figure 1*E*). We therefore adopted the minimal therapeutic dose of 1 μg/mL of Nal for all other experiments.

Treatment of infection with 1 μg/mL of Nal prevented host death in response to low-dose (Figure 1*B*, *C*) and high-dose (Supplementary Figure 1*D, E*) infections. It also inhibited bacterial growth in both cases (Figure 1*B*, Supplementary Figure 1*E*); therefore, larvae did not experience an acute phase of infection vs untreated larvae but instead immediately progressed toward a clearing phase (Figure 1*B*, Supplementary Figure 1*A–C*). In the presence of 1 μg/mL of Nal, the rate of bacterial clearance was not significantly different from that of untreated larvae, where infection was not fully cleared and had progressed toward a persistent phase. To investigate if bacteria infecting zebrafish in the persistent phase had developed antibiotic tolerance, irrespective of being treated with an antibiotic, we treated larvae exhibiting acute and persistent *Shigella* infection with 1 μg/mL of Nal for 24 hours and found that treatment significantly decreased bacterial burden approximately 100-fold during acute infection (Figure 1*D*). In contrast, bacterial burden in larvae in the persistent phase did not significantly decrease with antibiotic treatment. At 72 hpi, we observed that spontaneous evolution of antibiotic resistance occurred in 0.53% of larvae undergoing antibiotic treatment, while it was not observed in untreated larvae (Supplementary Figure 1*G*). This indicates that failure of 1 μg/mL of Nal to clear persistent bacteria in most larvae (99.47%) results from antibiotic tolerance.

**Figure 2.**
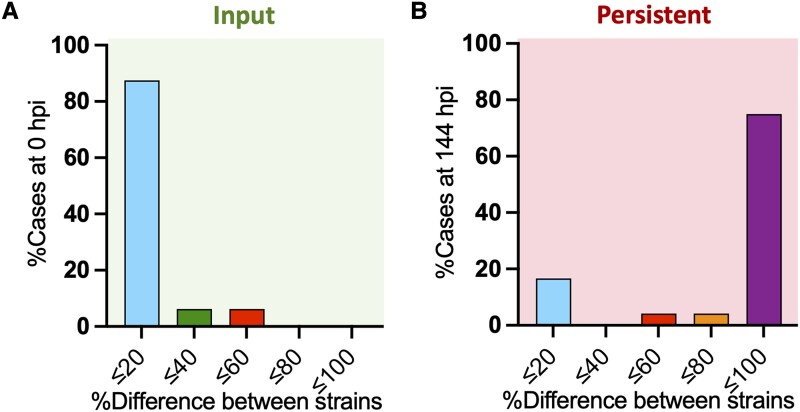
Persistent *Shigella* represents a clonally expanded population. mCherry- and GFP-labeled *S. sonnei* 53G strains were coinjected at a 1:1 ratio. *A*, At 0 hpi, most larvae (approximately 90%) displayed an expected 1:1 ratio of the coinjected strains (ie, percentage difference between strains ≤20%). *B*, At 144 hpi, most larvae (approximately 80%) were infected by 1 of the 2 strains at a much higher frequency than the other strain (ie, percentage difference between strains >80%), indicating the establishment of clonality. Statistics: differences in distribution between data at 0 and 144 hpi were calculated by chi-square test. ****P < .0001. hpi, hours postinfection.

To study whether persistent bacteria represent a clonally expanded population or derive from a stochastic reduction of the bacterial load, we coinjected *S. sonnei* 53G labeled with GFP or mCherry (and otherwise isogenic) at a 1:1 ratio (Figure 2). As expected, at 0 hpi, both strains could be recovered from most larvae (approximately 90%) at similar loads (difference in the recovery of the 2 strains ≤20%). Strikingly, at 144 hpi, most larvae (approximately 80%) dominantly carried only 1 of the 2 strains (difference in the recovery of the 2 strains >80%). These results indicate that persistent *Shigella* infections represent a clonal population.

Together, these data show that *Shigella* can establish persistent infection in vivo, characterized by poor host clearance over an extended period, antibiotic tolerance, and clonality.

### *Shigella* O-Ag Variants Circulating in the MSM Community Are Associated With Persistent Infection In Vivo

**Figure 3.**
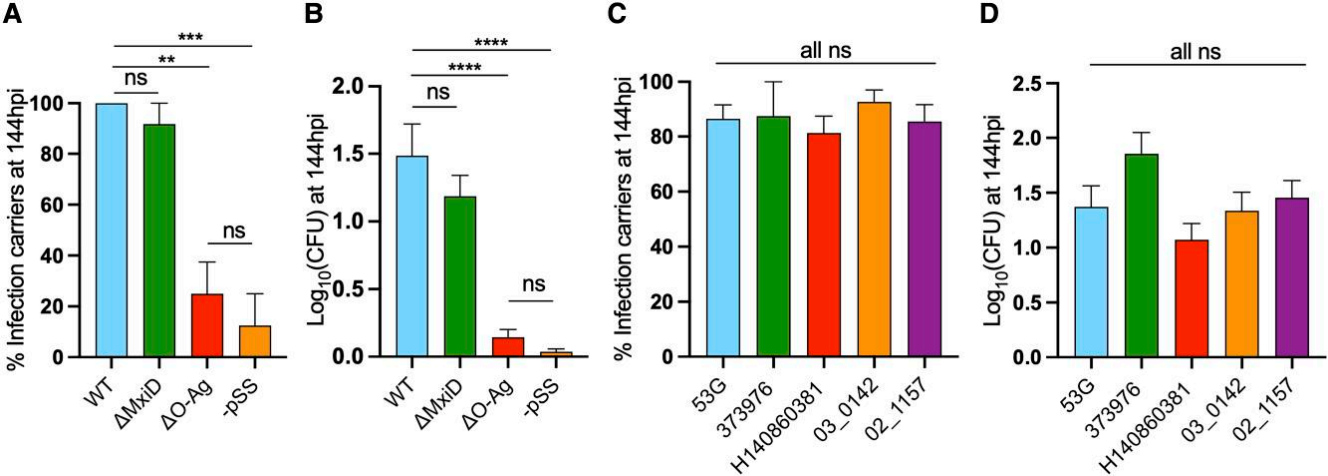
*Shigella* O-antigen is essential to establish persistent infection. *A* and *B*, Percentage of larvae carrying persistent infection at 144 hpi and average bacterial log_10_CFU for wild type *S. sonnei* 53G and several isogenic mutants. A T3SS mutant (ΔMxiD) can establish persistent infection at similar levels as wild type. However, the –pSS mutant (depleted of the virulence plasmid) and an O-antigen mutant (ΔO-Ag) are significantly reduced in their ability to establish persistent infections. *C* and *D*, Percentage of larvae carrying persistent infection at 144 hpi and average bacterial log_10_CFU for several *S. sonnei* isolates from lineage II and lineage III. Lineage II and III *S. sonnei* isolates are both capable of establishing persistent infections. Although belonging to different lineages, all *S. sonnei* isolates share an identical O-antigen. Data: mean ± SEM. Statistics: 1-way analysis of variance with Sidak correction on (*A*, *C*) percentage data or (*B*, *D*) log_10_-transformed data. ns, *P* ≥ .05. **P < .01. ***P < .001. ****P < .0001. CFU, colony-forming unit; hpi, hours postinfection; ns, nonsignificant; T3SS, type III secretion system; WT, wild type.

To investigate bacterial factors required to persist in vivo, we performed infections using various *S. sonnei* mutants, including a type III secretion system–deficient strain (ΔMxiD), an O-Ag–deficient strain (ΔO-Ag), and a strain having lost the virulence plasmid (–pSS). We observed that the type III secretion system–deficient strain could establish persistent infection in vivo, although the O-Ag–deficient and –pSS strains could not (Figure 3*A*, *B*; Supplementary Figure 2*A, B*). Considering that the O-Ag of *S. sonnei* is encoded by the pSS plasmid and that –pSS strains are O-Ag deficient, we conclude that O-Ag and not type III secretion system is essential for *Shigella* persistent infection. In agreement, we tested other isolates of *S. sonnei* (all iso-serotypic), including recently collected clinical isolates. While host survival rates vary upon infection with different isolates, all strains established persistent infections at similar levels (Figure 3*C*, *D*; Supplementary Figure 2*C, D*).

**Figure 4.**
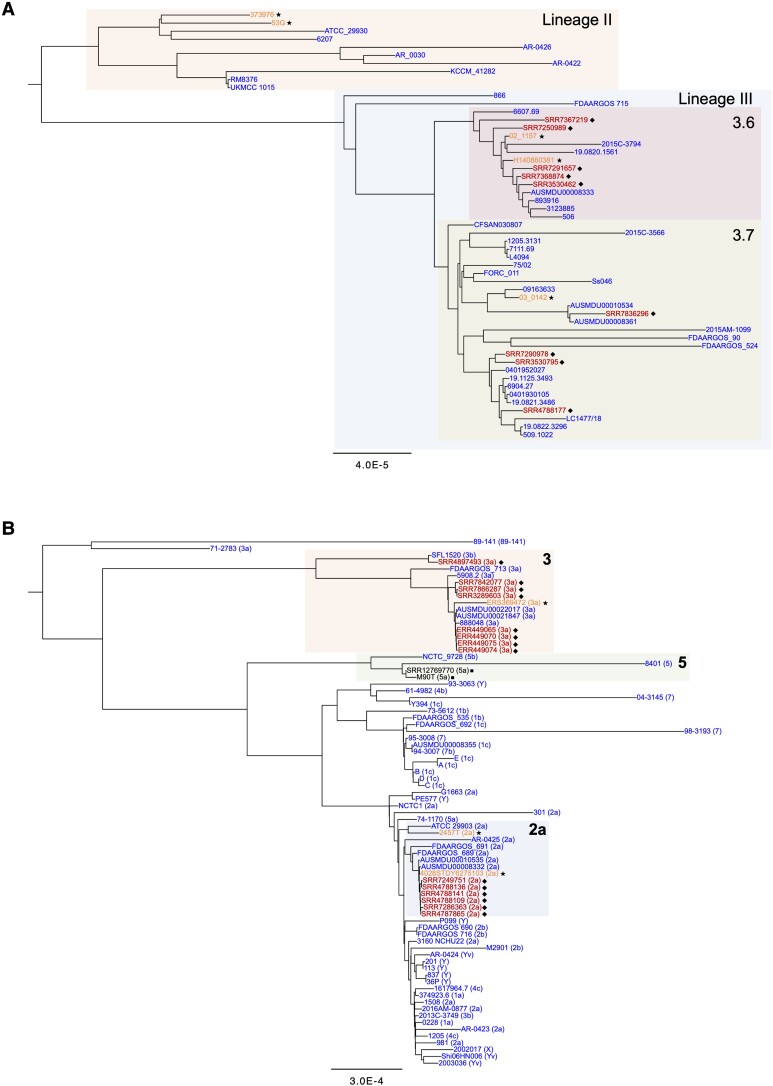
Phylogenetic distribution of persistent *Shigella.* Phylogenetic trees (codon trees) for (*A*) *S. sonnei* and (*B*) *S. flexneri* were constructed by using complete reference genomes of known serotypes (available in BV-BRC, https://www.bv-brc.org/), draft genome sequences of isolates associated with persistent carriage in humans [29], and the isolates used in this study (sequenced with nanopore technology). Red (labeled with diamond symbols ◆), strains associated with persistent carriage in humans; orange (labeled with star symbols ★), strains that established persistent infection in zebrafish; black (labeled with square symbols ■), strains that did not establish persistent infection in zebrafish; blue (unlabeled), other strains available in the BV-BRC database and utilized to construct the phylogenetic trees. For the *S. sonnei* tree, lineages II and III and sublineages 3.6 and 3.7 are highlighted. For the *S. flexneri* tree, the 3 main clusters encompassing the serotypes investigated in this study (2a, 3, and 5) are highlighted, and the serotype of each strain is reported in parentheses after the strain name. BV-BRC, Bacterial and Viral Bioinformatics Resource Centre.

More than 40 serotypes of *Shigella* have been reported, and O-Ag structure/composition is a major variable in determining these different serotypes [30]. Only 3 serotypes have been directly associated with carriage in the MSM community [5, 29]. To test whether strain clusters associated with carriage in humans establish persistent infection in zebrafish, we constructed phylogenetic trees for *S. sonnei* and *S. flexneri* using high-quality complete genomes deposited in the BV-BRC database, sequencing data collected from cases of likely carriage [29], and newly collected sequencing data from *S. sonnei* and *S. flexneri* clinical isolates selected for further testing in zebrafish (Figure 4). For the construction of trees, we used the codon tree method and the phylogenetic tree–building service made available by BV-BRC [27]. On the basis of this phylogenetic analysis, we selected *Shigella* isolates that represented either a persistent infection [20] ingroup (ie, falling in a genetic cluster that encompasses isolates previously associated with carriage in humans [29]) or a persistent infection outgroup (ie, not directly clustering with isolates previously associated with carriage in humans) for zebrafish infection.

**Figure 5.**
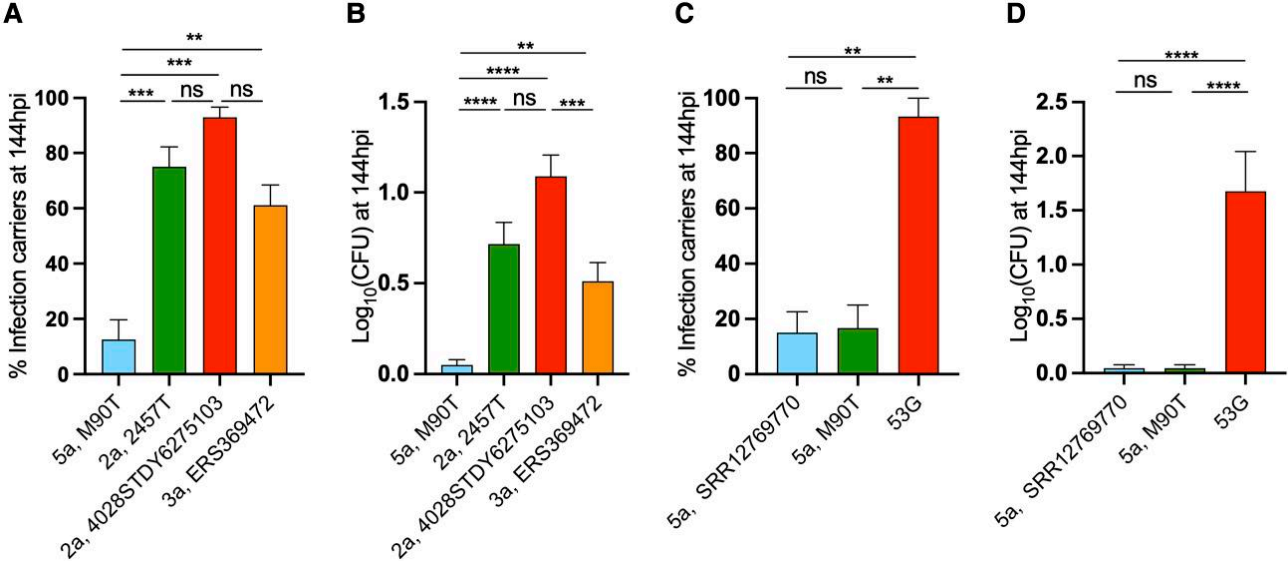
*Shigella* O-antigen serotypes associated with MSM transmission enable persistent infection. *A* and *B*, Percentage of larvae carrying persistent infection at 144 hpi and average bacterial log_10_CFU for several *S. flexneri* isolates from serotypes 2a, 3a, and 5a. *S. flexneri* isolates of serotypes 2a and 3a are both capable of establishing persistent infection, to a much greater extent than *S. flexneri* of serotype 5a. *C* and *D*, Percentage of larvae carrying persistent infection at 144 hpi and average bacterial log_10_CFU for *S. flexneri* M90T (serotype 5a), *S. sonnei* 53G, and *S. flexneri* SRR12769770 (a 2020 clinical isolate of *S. flexneri* from serotype 5a). SRR12769770 does not show a significant difference in the establishment of persistent infection when compared with *S. flexneri* M90T. Data: mean ± SEM. Statistics: 1-way analysis of variance with Sidak correction on (*A*, *C*) percentage data or (*B*, *D*) log_10_-transformed data. ns, *P* ≥ .05. **P < .01. ***P < .001. ****P < .0001. CFU, colony-forming unit; hpi, hours postinfection; MSM, men who have sex with men; ns, nonsignificant.

For *S. sonnei*, different sublineages have been identified [2, 31, 32]; however, only 1 *S. sonnei* serotype exists (Figure 4*A*), which has comparable genetic diversity to individual serotypes of *S. flexneri* (eg, *S. flexneri* 2a; Figure 4*B*) [33, 34]. Both representatives of the persistent infection ingroup and the persistent infection outgroup of *S. sonnei* persisted in zebrafish in vivo. This finding is not surprising, considering that *S. sonnei* isolates have short genetic distances and all share an identical O-Ag that defines the sole *S. sonnei* serotype (Figure 3*C*, *D*; Supplementary Figure 2*C*, *D*). In the case of *S. flexneri*, multiple serotypes have been identified [35], although only 2 serotypes (2a and 3a) have been so far associated with persistent carriage [5] (Figure 4*B*). Strikingly, representative strains of these serotypes also persisted in zebrafish (Figure 5; Supplementary Figure 3*A, B*). In contrast, 2 isolates belonging to the 5a serotype—*S. flexneri* M90T and SRR12769770, representing a persistent infection outgroup—were unable to persist in zebrafish. Overall, these results indicate that O-Ag type is associated with the establishment of persistent infection. Our findings are consistent with epidemiologic evidence showing that transmission in the MSM community is associated with *S. sonnei*, *S. flexneri* 2a, and *S. flexneri* 3a serotypes but not *S. flexneri* 5a serotype [5].

### *Shigella* Can Establish Persistent Infection in Macrophages In Vivo

**Figure 6.**
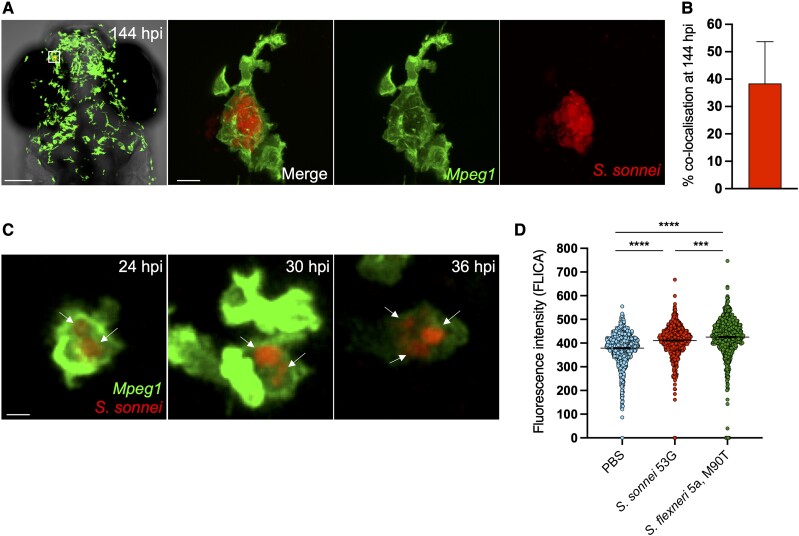
*Shigella* can establish persistent infection of macrophages in vivo. *A*, Head region and individual macrophage detail from a representative *S. sonnei*–infected zebrafish larva at 144 hpi. *Tg(mpeg1::Gal4-FF)*^gl25^/*Tg(UAS::LIFEACT-GFP)*^mu271^ larvae (Mpeg1, with macrophages in green) were injected at 3 dpf in the hindbrain ventricle with 1000 CFU of mCherry-labeled *S. sonnei* 53G (red). An infected macrophage harboring persistent bacteria is magnified. Scale bars: 100 μm, left; 10 μm, right and inset. *B*, At the persistent infection stage, 40% of bacterial fluorescence colocalizes with *mpeg1^+^* macrophages. *C*, Individual *Tg(mpeg1::Gal4-FF)*^gl25^/*Tg(UAS::LIFEACT-GFP)*^mu271^ macrophage (Mpeg1, green) harboring mCherry–*S. sonnei* (red, indicated by arrows) followed for 12 hours (from 24 to 36 hpi). At 2 dpf, 1000 CFU of bacteria were delivered systemically in larvae. Scale bar: 10 μm. *D*, As compared with *S. flexneri* M90T, *S. sonnei* 53G induces a reduced level of caspase 1 activation in vivo in the zebrafish model. At 3 dpf, 10000 CFU of bacteria were delivered systemically in larvae. Data: mean ± SEM. Statistics: 1-way analysis of variance with Sidak correction. ***P < .001. ****P < 0.0001. CFU, colony-forming unit; dpf, days postfertilization; hpi, hours postinfection; PBS, phosphate-buffered saline.

Macrophages are viewed as a first line of host defense against *Shigella* infection and have been reported to act as a long-term reservoir for intracellular bacterial pathogens, such as *S. enterica* and *M. tuberculosis* [13, 14, 36]. Having established a zebrafish model of *Shigella* persistent infection, we sought to determine whether this was localized to macrophages. Using the *Tg(mpeg1::Gal4-FF)*^gl25^/*Tg(UAS::LIFEACT-GFP)*^mu271^ transgenic zebrafish line labeling macrophages and mCherry-labeled *S. sonnei* 53G, we identified approximately 40% of bacterial fluorescence colocalizing with macrophages at 144 hpi (Figure 6*A*, *B*; Supplementary Video 1). Longitudinal studies with high-resolution confocal microscopy showed that macrophages harboring a stable bacterial load (ie, bacterial fluorescence showing a fold change of only 1.21 ± 0.21 in 12 hours) can be detected as early as 24 hpi (Figure 6*C*, Supplementary Video 2) and last for several consecutive days (Supplementary Figure 4*A–C*). These data were surprising, considering that macrophage cell death is widely recognized as a hallmark of *Shigella* infection [17, 37, 38]. *Shigella* establishing persistent infection inside macrophages did not maintain the classical rod shape, which can be observed before phagocytosis and at early time points following macrophage invasion (eg, at 4 hpi; Figure 6*A*; Supplementary Figure 4*D, E*) [22].

Previous work indicated that *S. sonnei* may be less efficient than *S. flexneri* M90T (5a) in inducing macrophage cell death in vitro [38]. We hypothesized that the reduced ability to induce macrophage death may explain the ability to establish the persistent infection of macrophages. To test this in vivo, we infected zebrafish larvae with *S. sonnei* 53G or *S. flexneri* 5a M90T systemically via an infection route that promotes *Shigella*-macrophage interactions [39, 40]. At 4 hpi, we dissociated infected larvae and PBS injection control larvae and quantified by flow cytometry the level of caspase 1 activity, a marker of macrophage pyroptotic cell death (Figure 6*D*). Considering that this assay measures differences in caspase 1 activity at the whole macrophage population level, differences between groups appear small because only a fraction of the macrophage population is infected. Despite this limitation, infection with *S. flexneri* 5a M90T led to significantly higher levels of caspase 1 activity as compared with *S. sonnei* 53G, consistent with the possibility that persistent *Shigella* serotypes elicit significantly less macrophage death in vivo than serotypes unable to establish persistent infections.

## DISCUSSION

The zebrafish infection model has been instrumental to study host-pathogen interactions in response to a variety of human pathogens, including different *Shigella* [22, 41–43]. Here, we show that different *Shigella* serotypes (*S. sonnei*, *S. flexneri* 2a and 3a) that are highly prevalent in the MSM community and associated with direct host-to-host transmission also establish persistent infection in zebrafish. In contrast, the *S. flexneri* 5a serotype, which is less prevalent in the MSM community, is unable to establish persistent infection in vivo. There are likely multiple factors that may explain why the *S. flexneri* 5a serotype is less prevalent in the MSM community and worldwide. Given our evidence, we propose that the inability of the *S. flexneri* 5a serotype to persist could be a significant factor underlying its replacement with more prevalent serotypes able to establish persistent infections.

Our results show that O-Ag variants promote the establishment of persistent infection, but it is not yet known how the O-Ag precisely contributes. *S. sonnei* O-Ag is unique among enterobacterial pathogens and does not share obvious structural homology with the O-Ag expressed by *S. flexneri* 2a or 3a. However, *Shigella* species are a remarkable example of convergent evolution [33], and the persistent phenotype mediated by different O-Ag serotypes may represent an example of pathoadaptive convergence due to occupation of the same niche. During the persistent infection phase, bacteria frequently colocalize with macrophages in vivo, suggesting that macrophages represent a preferred niche for *Shigella* persistent infection. *Shigella* establishing persistent infection often assumed pleomorphic shapes and lost their rod shape following macrophage invasion. This is in agreement with previous work demonstrating that several bacterial species transition to L-forms within macrophages (eg, in response to lysozyme [44]) and during zebrafish infection [45].

Isolates of *S. sonnei* serotype, which is associated with persistent infection, induce significantly less macrophage death in vivo as compared with isolates of *S. flexneri* serotype 5a, which has not been linked to persistent infection and MSM transmission. Considering that persistent infection of zebrafish recapitulates the epidemiologic trend of carriage in humans, we propose that zebrafish can be used to discover host and pathogen factors underlying *Shigella* persistent infection in humans.

## Supplementary Data

Supplementary materials are available at *The Journal of Infectious Diseases* online. Consisting of data provided by the authors to benefit the reader, the posted materials are not copyedited and are the sole responsibility of the authors, so questions or comments should be addressed to the corresponding author.

## Supplementary Material

jiad326_Supplementary_Data

## Notes

***Acknowledgments.*** We thank the Wiebke Herzog laboratory for the *Tg(UAS::LIFEACT-GFP)*^mu271^ zebrafish line and all members of the Mostowy, Baker, and Holt laboratories for discussion and experimental help.

***Author contributions.*** This project was conceived by V. T. and S. M., V. T. and S. L. M. performed all zebrafish experiments and analyzed all data sets. V. T., S. L. M., C. E. C., and P. M. D. S. prepared DNA samples and analyzed sequencing results. V. T. constructed the phylogenetic trees with inputs from K. E. H., S. L. M., and K. S. B., V. T. and D. B. performed experiments to detect active caspase 1 in zebrafish. K. S. B. and C. J. provided valuable strains for the work. V. T. and S. M. wrote the manuscript with input from all authors.

***Data availability*.**  *Shigella* genetically modified strains generated in this study are available upon request. Sequencing data generated in this study have been deposited in the Sequencing Read Archive at BioProject PRJNA869897 and are publicly available as of the date of publication. Accession numbers are listed against individual strains in Supplementary Table 1.

***Financial support*.** V. T. was supported by a London School of Hygiene and Tropical Medicine/Wellcome Trust Institutional Strategic Support Fund fellowship (204928/Z/16/Z). S. L. M. is supported by a PhD studentship (BB/T008709/1) from the London Interdisciplinary Doctoral Programme, Biotechnology and Biological Sciences Research Council. D. B. is supported by the Deutsche Forschungsgemeinschaft Walter Benjamin Programme (BR 6637/1-1). Research in the laboratory of K. S. B. is supported by an Academy of Medical Sciences Springboard award (SBF002\1114), a Medical Research Council New Investigator award (MR/R020787/1), and the Biotechnology and Biological Sciences Research Council (BB/V009184/1). Research in the laboratory of S. M. is supported by a European Research Council Consolidator Grant (772853; ENTRAPMENT), Wellcome Trust Senior Research Fellowship (206444/Z/17/Z), and the Lister Institute of Preventive Medicine. Funding to pay the Open Access publication charges for this article was provided by the London School of Hygiene and Tropical Medicine.
