# Supplementary material for: Shigella Serotypes Associated With Carriage in Humans Establish Persistent Infection in Zebrafish
Source: J Infect Dis. 2023 Aug 9;228(8):1108–18. doi: 10.1093/infdis/jiad326 (PMC10582909; doi:10.1093/infdis/jiad326)

Supplementary Figure 1 (related to Fig. 1). *Shigella* establishes persistent infections in zebrafish

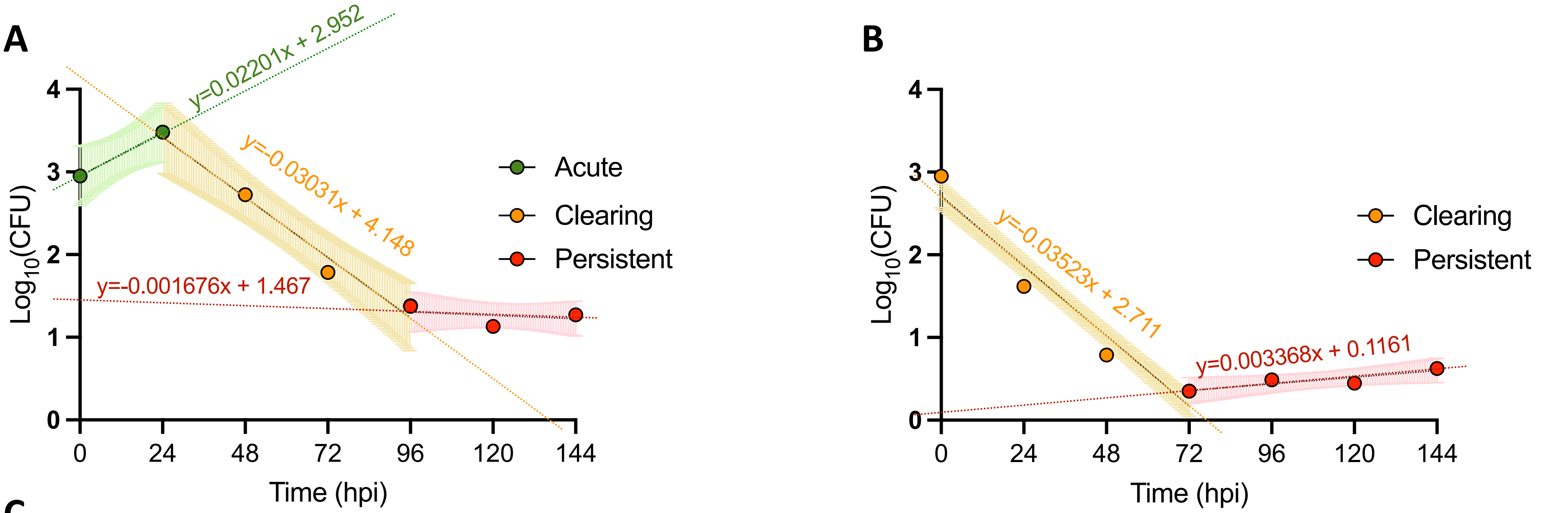

C

	Untreated Acute	Untreated Clearing	Untreated Persistent	1 µg/ml Nal Clearing	1 µg/ml Nal Persistent
Untreated Clearing	p=0.0005	-	-	-	-
Untreated Persistent	p=0.0200	p<0.0001	-	-	-
1 µg/ml Nal Clearing	p<0.0001	ns p=0.3165	p<0.0001	-	-
1 µg/ml Nal Persistent	p=0.0204	p<0.0001	ns p=0.1750	p<0.0001	-

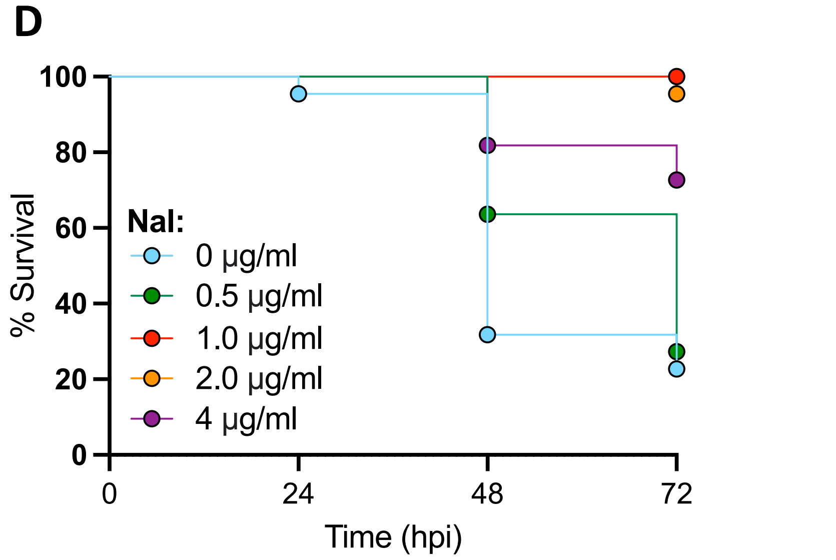

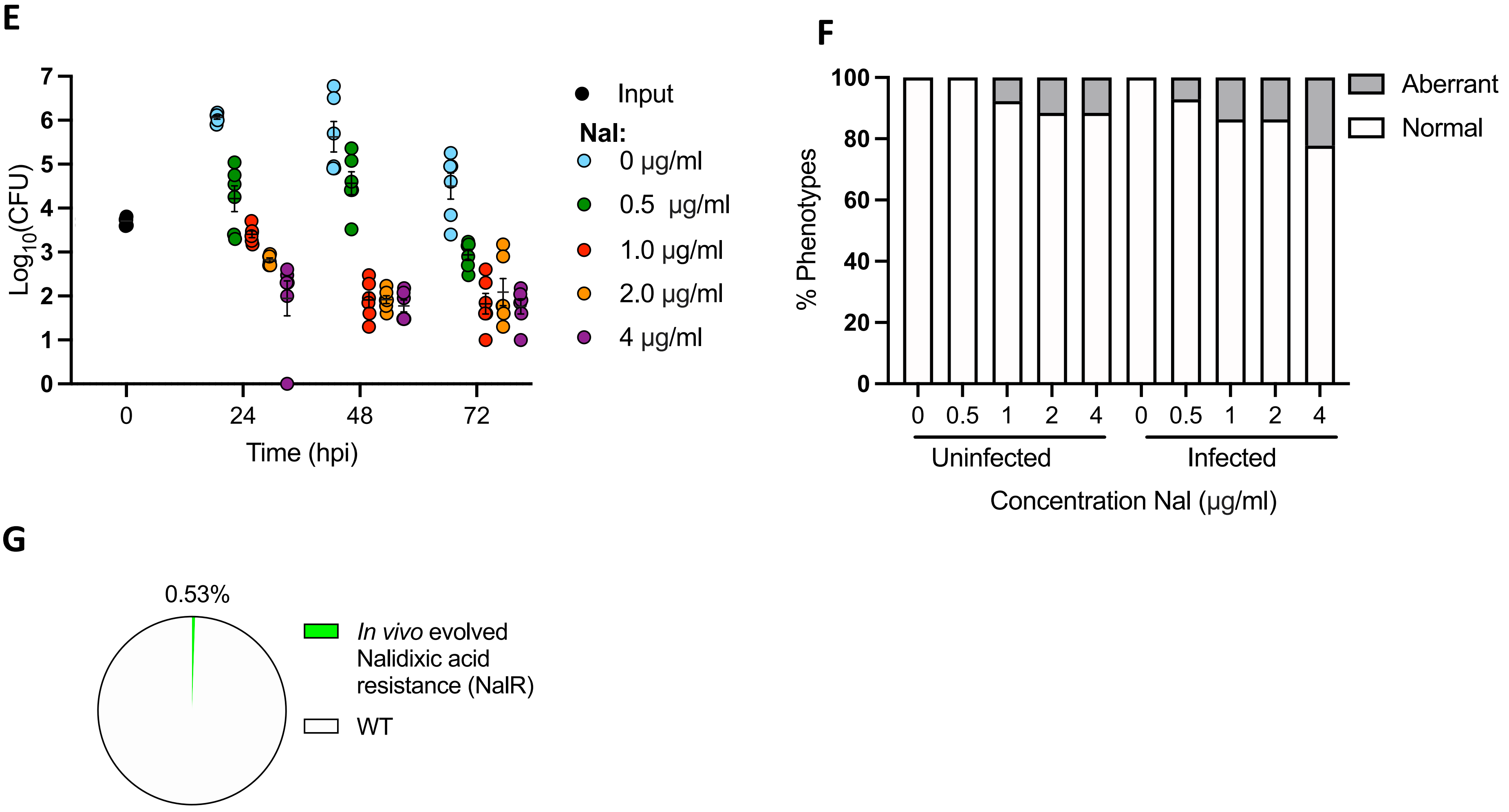

Supplement: jiad326_Supplementary_Data [file jiad326_supplementary_data.zip › Supplementary Figure 1.pdf]
