# Supplementary material for: Shigella Serotypes Associated With Carriage in Humans Establish Persistent Infection in Zebrafish
Source: J Infect Dis. 2023 Aug 9;228(8):1108–18. doi: 10.1093/infdis/jiad326 (PMC10582909; doi:10.1093/infdis/jiad326)

Supplementary Figure 2 (related to Fig. 3). *Shigella* O-Antigen is essential to establish persistent infection

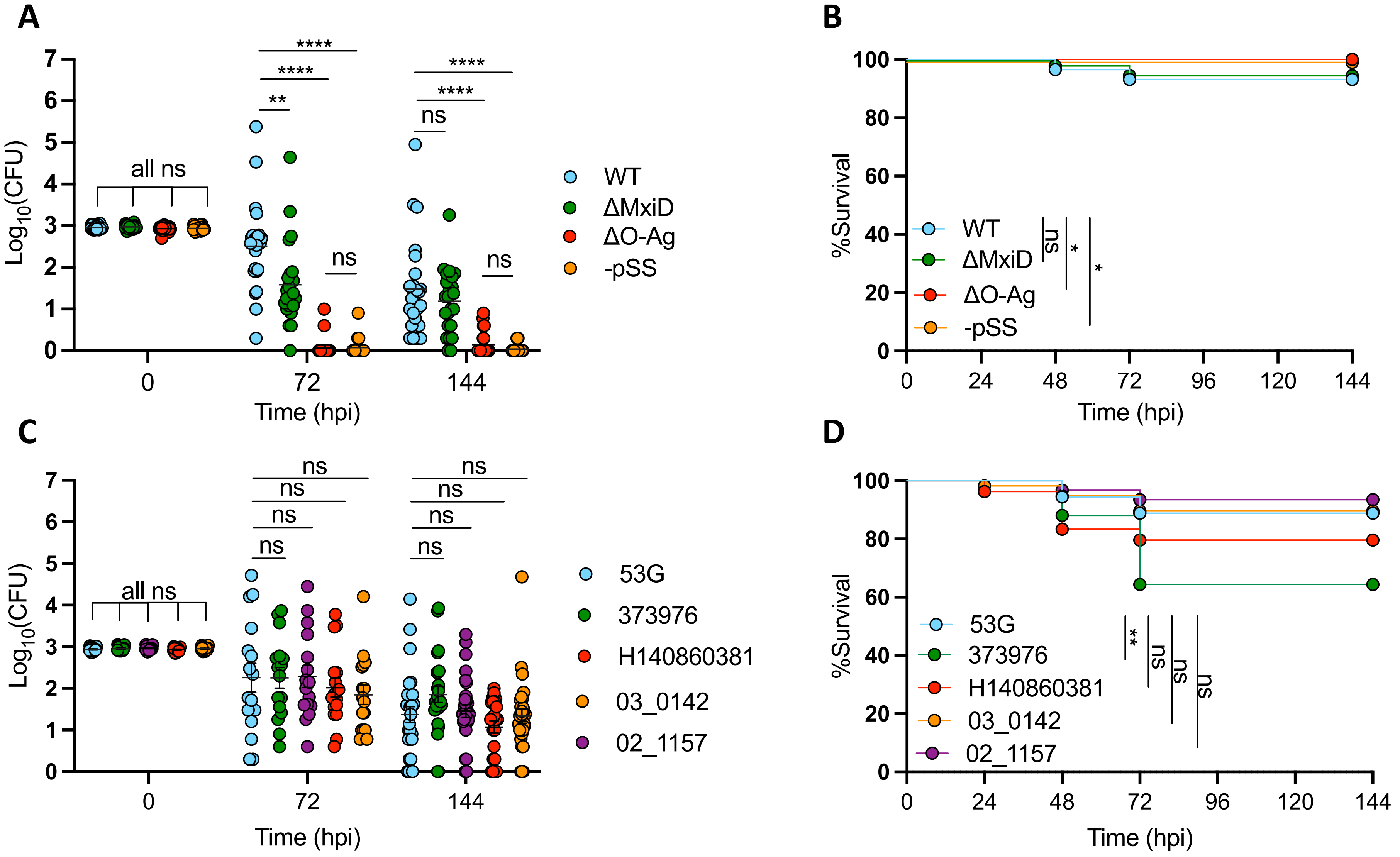

Supplement: jiad326_Supplementary_Data [file jiad326_supplementary_data.zip › Supplementary Figure 2.pdf]
