# Supplementary material for: Shigella Serotypes Associated With Carriage in Humans Establish Persistent Infection in Zebrafish
Source: J Infect Dis. 2023 Aug 9;228(8):1108–18. doi: 10.1093/infdis/jiad326 (PMC10582909; doi:10.1093/infdis/jiad326)

Supplementary Figure 4 (related to Fig. 6). *Shigella* can establish persistent infection of macrophages *in vivo*

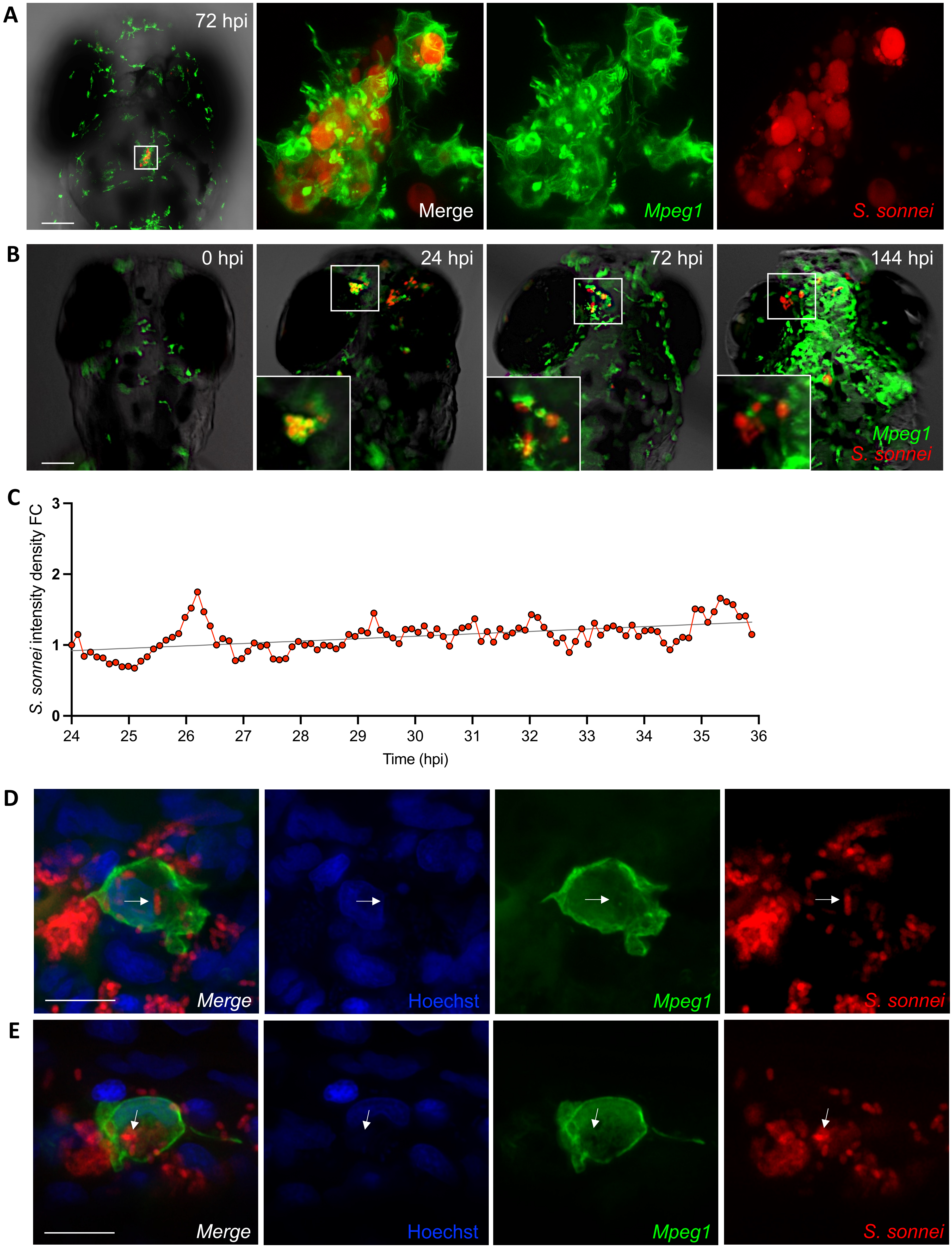

Supplement: jiad326_Supplementary_Data [file jiad326_supplementary_data.zip › Supplementary Figure 4.pdf]
