# Supplementary material for: Shigella Serotypes Associated With Carriage in Humans Establish Persistent Infection in Zebrafish
Source: J Infect Dis. 2023 Aug 9;228(8):1108–18. doi: 10.1093/infdis/jiad326 (PMC10582909; doi:10.1093/infdis/jiad326)
Supplement: jiad326_Supplementary_Data [file jiad326_supplementary_data.zip › SUPPLEMENTARY MATERIAL LEGENDS.docx]

**SUPPLEMENTARY MATERIAL LEGENDS**

**Supplementary Figure 1 (related to Fig. 1). *Shigella* establishes persistent infection in zebrafish.**

(**A-C**) Linear regression analysis of the change of Log_10_(CFU) of *S. sonnei* 53G as a function of time for untreated larvae (A) or larvae treated with the antibiotic Nalidixic acid (B), and relevant summary statistics (C). In the absence of antibiotics, *Shigella* infection progresses in three distinct phases (acute, clearing, and persistent), that can be described by individual linear equations. Treatment with therapeutic doses of Nalidixic acid prevents infection from undergoing acute expansion, but it does not promote complete eradication of the infection. The presence or absence of the antibiotic does not significantly impact the trend (slope) of Log_10_(CFU) over time, neither during the clearing phase nor during the persistence phase. (**D-F**) Survival curves (D), Log_10_(CFU) (E) and phenotypic analysis (F) of zebrafish larvae infected with a lethal dose (i.e., 8000 CFU) of *S. sonnei* 53G and treated with different concentrations of Nalidixic acid. 1 μg/ml Nalidixic acid was sufficient to completely rescue host survival (D) and prevent significant bacterial growth *in vivo* during the acute infection phase (E). Toxicity at this antibiotic concentration is negligible (F) and <10% of treated larvae displayed aberrant phenotypes (i.e., cardiac oedemas). (**G**) *Shigella* persistent infection is associated with antibiotic tolerance. At 72 hpi (i.e., end of the clearing phase) only 0.53% of the tested larvae carried bacteria that developed an inheritable decreased susceptibility to the antibiotic treatment (i.e., 4xMIC).

**Supplementary Figure 2 (related to Fig. 3). *Shigella* O-Antigen is essential to establish persistent infection.**

(**A,B**) Log_10_CFU of individual larvae (A) and survival curves (B), for WT *S. sonnei* 53G and several isogenic mutants. (**C,D**) Log_10_CFU of individual larvae (C) and survival curves (D) for several *S. sonnei* isolates from Lineage II and Lineage III. Statistics: one-way ANOVA with Sidak’s correction on Log_10_-transformed data (A,C); Log-rank Mantel-Cox test (B,D); ns (non-significant) p≥0.05; *p<0.05; **p<0.01; ****p<0.0001.

**Supplementary Figure 3 (related to Fig. 5). Different *Shigella* O-Antigen serotypes associated with MSM transmission enable persistent infection.**

(**A,B**) Log_10_CFU of individual larvae (A) and survival curves (B) for several *S. flexneri* isolates from serotypes 2a, 3a and 5a. Statistics: one-way ANOVA with Sidak’s correction on Log10-transformed data (A); Log-rank Mantel-Cox test (B); ns (non-significant) p≥0.05; ***p<0.001; ****p<0.0001.

**Supplementary Figure 4 (related to Fig. 6). *Shigella* can establish persistent infection of macrophages *in vivo.***

(**A**) Head region and individual macrophage detail from a representative *S. sonnei-*infected zebrafish larvae at 72 hpi. *Tg(mpeg1::Gal4-FF)*^gl25^/*Tg(UAS::LIFEACT-GFP)*^mu271^ larvae (Mpeg1, with macrophages in green) were injected at 3 dpf in the hindbrain ventricle with 1,000 CFU mCherry-labelled *S. sonnei* 53G (red). A cluster of infected macrophages harbouring persistent bacteria is magnified. Scalebars: 100 μm (left); 10 μm (right, inset). (**B**) Longitudinal imaging of the head region from a representative *S. sonnei-*infected zebrafish larvae at 0, 24, 72 and 144 hpi. The same region of interest is magnified in the bottom left corner. Scalebar: 100 μm. (**C**) Longitudinal quantification of bacterial fluorescence in an individual macrophage harbouring mCherry-*S. sonnei,* followed over time for 12 h (from 24 to 36 hpi). The bacterial fluorescence remains overall constant or only slightly increases (i.e., bacterial fluorescence shows only a 1.21±0.21 fold change over a 12 h period of observation). For data in C, 1000 CFU bacteria were delivered systemically in 2 dpf larvae. (**D,E**). Representative images of *Tg(mpeg1::Gal4-FF)*^gl25^/*Tg(UAS::LIFEACT-GFP)*^mu271^ macrophages (Mpeg1, green) infected with mCherry-labelled *S. sonnei* 53G (red) at 4 hpi. DNA was labelled with Hoechst staining (blue). At early time points, *Shigella* can be seen inside macrophages with a classical rod shape (arrow in D). However, at this time point, some bacteria can already be seen to transition from rod-shaped to a pleomorphic shape (arrow in E). For images in D-E, 10000 CFU bacteria were delivered systemically in 2 dpf larvae. Scalebar: 10 μm.

**Supplementary Table 1. Bacterial strains used in this study.**

**Supplementary Table 2. Details of newly sequenced/re-sequenced bacterial strains used in this study.**

**Supplementary Video 1 (related to Fig. 6). Confocal z-stack of a macrophage carrying *S. sonnei* infection at 144 hpi.**

*Tg(mpeg1::Gal4-FF)*^gl25^/*Tg(UAS::LIFEACT-GFP)*^mu271^ larvae (with macrophages labelled in green) were injected at 3 dpf in the hindbrain ventricle with 1000 CFU mCherry-labelled *S. sonnei* 53G (red). Images were taken by confocal microscopy at 144 hpi.

**Supplementary Video 2 (related to Fig. 6). Confocal time-lapse of a macrophage carrying *S. sonnei* infection for 12h.**

*Tg(mpeg1::Gal4-FF)*^gl25^/*Tg(UAS::LIFEACT-GFP)*^mu271^ larvae (with macrophages labelled in green) were injected at 2 dpf via the caudal vein with 1000 CFU mCherry-labelled *S. sonnei* 53G (red). Images were taken by confocal microscopy at 6.5 min intervals for 12 h, from 24 to 36 hpi.

**Supplementary Dataset (related to Fig. 1). Raw CFU counts from data in Figure 1B,D.**
