# Supplementary material for: Shigella Serotypes Associated With Carriage in Humans Establish Persistent Infection in Zebrafish
Source: J Infect Dis. 2023 Aug 9;228(8):1108–18. doi: 10.1093/infdis/jiad326 (PMC10582909; doi:10.1093/infdis/jiad326)
Supplement: jiad326_Supplementary_Data [file jiad326_supplementary_data.zip › Supplementary Table 2.docx]

**Supplementary Table 2. Genetically modified bacterial strains used in this study**

| **Species** | **Serotype** | **Strain background** | **Reporter construct** | **Mutations** | **Reference** |
| --- | --- | --- | --- | --- | --- |
| *Shigella sonnei* | Ss | 53G | GFP from pFPV25.1 (Valdivia et al., 2006) | WT | Torraca et al., 2019 |
| *Shigella sonnei* | Ss | 53G | GFP from pFPV25.1 (Valdivia et al., 2006) | ΔMxiD (Watson et al., 2018) | Torraca et al., 2019 |
| *Shigella sonnei* | Ss | 53G | GFP from pFPV25.1 (Valdivia et al., 2006) | ΔO-Ag (Watson et al., 2019) | Torraca et al., 2019 |
| *Shigella sonnei* | Ss | 53G | GFP from pFPV25.1 (Valdivia et al., 2006) | -pSS (Torraca et al., 2019) | Torraca et al., 2019 |
| *Shigella sonnei* | Ss | 53G | mCherry from pFPV-mcherry (Drecktrah et al., 2008) | WT | Torraca et al., 2019 |
| *Shigella sonnei* | Ss | H140860381 | mCherry from pFPV-mcherry (Drecktrah et al., 2008) | WT | This study |
| *Shigella sonnei* | Ss | 03_0142 | mCherry from pFPV-mcherry (Drecktrah et al., 2008) | WT | This study |
| *Shigella sonnei* | Ss | 02_1157 | mCherry from pFPV-mcherry (Drecktrah et al., 2008) | WT | This study |
| *Shigella flexneri* | 5a | M90T | mCherry from pFPV-mcherry (Drecktrah et al., 2008) | WT | This study |
| *Shigella flexneri* | 5a | SRR12769770 | mCherry from pFPV-mcherry (Drecktrah et al., 2008) | WT | This study |
| *Shigella flexneri* | 2a | 2457T | mCherry from pFPV-mcherry (Drecktrah et al., 2008) | WT | This study |
| *Shigella flexneri* | 2a | 4028STDY6275103 | mCherry from pFPV-mcherry (Drecktrah et al., 2008) | WT | This study |
| *Shigella flexneri* | 3a | ERS369472 | mCherry from pFPV-mcherry (Drecktrah et al., 2008) | WT | This study |
