# Supplementary figures and images for: Shigella Serotypes Associated With Carriage in Humans Establish Persistent Infection in Zebrafish
Source: J Infect Dis. 2023 Aug 9;228(8):1108–18. doi: 10.1093/infdis/jiad326 (PMC10582909; doi:10.1093/infdis/jiad326)

**B**

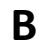

Supplement: jiad326_Supplementary_Data [file jiad326_supplementary_data.zip › Supplementary Figure 3.pdf]
